# Grizzly bear response to fine spatial and temporal scale spring snow cover in Western Alberta

**DOI:** 10.1371/journal.pone.0215243

**Published:** 2019-04-10

**Authors:** Ethan E. Berman, Nicholas C. Coops, Sean P. Kearney, Gordon B. Stenhouse

**Affiliations:** 1 Department of Forest Resources Management, University of British Columbia, Vancouver, British Columbia, Canada; 2 fRI Research, Hinton, Alberta, Canada; Sichuan University, CHINA

## Abstract

Snow dynamics influence seasonal behaviors of wildlife, such as denning patterns and habitat selection related to the availability of food resources. Under a changing climate, characteristics of the temporal and spatial patterns of snow are predicted to change, and as a result, there is a need to better understand how species interact with snow dynamics. This study examines grizzly bear (*Ursus arctos*) spring habitat selection and use across western Alberta, Canada. Made possible by newly available fine-scale snow cover data, this research tests a hypothesis that grizzly bears select for locations with less snow cover and areas where snow melts sooner during spring (den emergence to May 31^st^). Using Integrated Step Selection Analysis, a series of models were built to examine whether snow cover information such as fractional snow covered area and date of snow melt improved models constructed based on previous knowledge of grizzly bear selection during the spring. Comparing four different models fit to 62 individual bear-years, we found that the inclusion of fractional snow covered area improved model fit 60% of the time based on Akaike Information Criterion tallies. Probability of use was then used to evaluate grizzly bear habitat use in response to snow and environmental attributes, including fractional snow covered area, date since snow melt, elevation, and distance to road. Results indicate grizzly bears select for lower elevation, snow-free locations during spring, which has important implications for management of threatened grizzly bear populations in consideration of changing climatic conditions. This study is an example of how fine spatial and temporal scale remote sensing data can be used to improve our understanding of wildlife habitat selection and use in relation to key environmental attributes.

## Introduction

Snow dynamics are a key driver of the seasonal behaviors of a variety of wildlife species, through influencing resource availability and fitness costs [1–3]. In landscapes with harsh seasonal conditions, snow cover can dictate food quality and distribution, and along with cold temperatures can result in patterns of hibernation and migration. For hibernators, the accumulation of snow in the fall and ablation in the spring have been linked to both spatial and temporal denning patterns [4–5]. Snow distribution can also adversely influence energy costs, through increased difficulty moving through a deep snowpack [6] and by dictating the timing of spring vegetation emergence [7–9].

Grizzly bear (*Ursus arctos)* populations in Alberta, Canada experience a long winter, characterized by persistent snow and freezing temperatures. The hibernation period lasts from around November to March [10], and the timing and location of denning has been linked to snow dynamics [11–12], with warm spring temperatures and reduced snow cover resulting in early den exit [5]. In addition, grizzly bears in Alberta den near high quality spring food resources [13], mainly in the form of sweet-vetch roots (*Hedysarum* spp.). During spring season, snow cover and frozen ground can restrict digging for sweet-vetch. Therefore bears have been shown to follow a “brown-tide”, progressively following the change from winter to spring conditions in search of optimal conditions for root-digging [14].

In a landscape dominated by anthropogenic presence and activity, changing snow dynamics due to climate change have the potential to increase the risk of negative human-bear encounters. Human-caused grizzly bear mortality is the most significant factor influencing bear population growth and long-term population sustainability [15–16]. In general, snow conditions in Western Alberta are spatially and temporally heterogeneous within a given winter season, and can vary markedly inter-annually [17]. Future climatic projections call for an increased uncertainty regarding the timing and extent of winter conditions [18], and an overall decrease in days with snow cover on an annual basis [19], especially during spring months [20]. In addition, climate models suggest higher and more frequent warmer temperatures during winter and spring, and a global average temperature rise of 1.5 ^o^C between the 20^th^ and 21^st^ century [21–22]. These changes in spring weather and snow patterns could lead to an earlier annual den emergence for grizzly bears, and could contribute to bears using lower elevations earlier in the year, since these locations are often the first to supply food resources. If vegetation phenology shifts do not match up with early snow melt, key food sources may not be available immediately after earlier den emergence. This could cause grizzly bears to use lower elevations for a prolonged period of time and range over more territory searching for food, thereby leading to increased human conflicts. However, if vegetation green-up shifts to match earlier snow-melt, grizzly bears may actually move to higher elevations earlier, in turn having a neutral effect or even reducing effect on human conflicts.

Although snow cover dynamics are largely unstudied in relation to grizzly bear spring habitat selection, a large body of work exists characterizing grizzly bear spring habitat selection in relation to a variety of other environmental and landscape variables. Elevation, solar insolation, topographic wetness, and landcover influence vegetation productivity and food availability and have been linked to grizzly bear habitat selection [23–25]. Bears also select for both natural and anthropogenic edges [26–27], related to an abundance of important habitat resources. Previous work has shown negative and positive selection for roads, due to both high food productivity and high risk [28–32]. Roads create forest edge habitat but also increase the risk of human-caused grizzly bear mortality, especially within 500 m of a road or 200 m of a trail [16,33]. Snow interacts with these variables and previous studies have commented on the potential influence of snow on spring habitat selection due to snow creating undesirable landscape conditions for bears [23,34–35]. Recent availability of fine-scale snow data may be key to better understanding spring selection [36].

By incorporating new fine-scale remote sensing snow cover data [37–38], this research seeks to build upon the existing body of knowledge surrounding the drivers of grizzly bear spring habitat selection and use in a region of core habitat in Western Alberta, Canada. We utilize Integrated Step Selection Analysis (iSSA) [39–42] to build a core model with variables previously shown to explain grizzly bear spring habitat selection and add snow cover data to see if it improves the accuracy of the model. Probability of use is then calculated to examine the average effect of snow cover, elevation, and distance to roads on grizzly bear habitat use. Elevation and distance to roads are key indicators of whether snow is driving bears to locations with higher risk of human encounters. Through this process we examine the hypothesis that bears are selecting for locations with lower percentages of snow cover during spring, and once snow has melted they are selecting for locations where it melted sooner on the landscape. In addition, snow cover data is used to analyze year-to-year variability in snow melt and how these trends may affect model accuracy. We aim to demonstrate the utility and flexibility of iSSA in examining and evaluating wildlife selection in response to spatially and temporally dynamic environmental variables derived from developments in remote sensing technology.

## Methods

In this section we first describe the study area and provide details on grizzly bear telemetry data, core model covariates, and snow covariates. We then describe the iSSA modelling approach, beginning with the development of a core model built using covariates previously shown to influence habitat selection during spring (Table 1). Snow covariates were added to the core model in three configurations to assess whether the inclusion of snow improved model accuracy. The best fitting model was evaluated to determine average effects of individual snow covariates on probability of use by grizzly bears.

**Table 1:** Covariates used in models and references to studies linking variables to grizzly bear habitat selection and use.

Covariate Name	Covariate Acronym	Spatial resolution	Temporal resolution	Previous work indicating relation to grizzly bear habitat selection and use
Natural log of step length	Ln(SL)	n/a	Hourly	[55]
Time of day	TOD	n/a	Hourly	[55]
Elevation	ELEV	30 m	Static	[23–25,34]
Distance to road	Dist(RD)	30 m	Static	[23,28–32,54]
Terrain wetness index	TWI	30 m	Static	[24]
Distance to forest edge	Dist(FE)	30 m	Yearly	[25–26,44,54,56,74,76]
Landcover	Landcover	30 m	Yearly	[23,55,75]
Solar insolation	INSOL	30 m	Static	[24–25,32,44]
Days since snow melt	DSM	30 m	Daily	[5,23,34–35]
Binary snow covered area	bSCA	30 m	Daily	[23,34–35]
Fractional snow covered area	fSCA	30 m	Daily	[23,34–35]

### Study area

**Fig 1:**
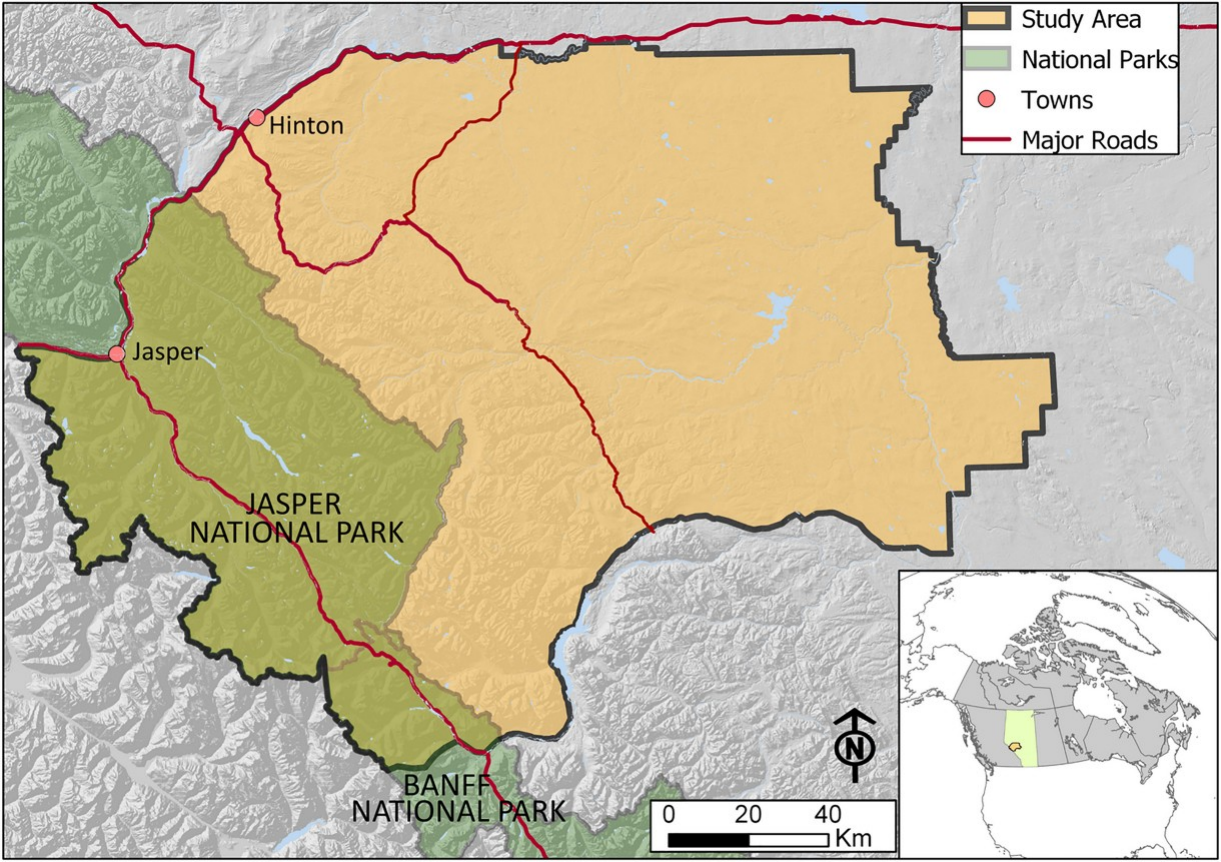
The Yellowhead bear management area (BMA3) in Western Alberta.

Our study area is a 28,529 km^2^ region of Western Alberta (52°91’ N, 116°69’W), which comprises the Yellowhead Bear Management Area (BMA3) (Fig 1). 30.5% of total area is designated as protected, mostly in Jasper National Park, with relatively low amounts of anthropogenic disturbance and activity [43]. The rest of the region, the foothills, is highly fragmented due to a history of fire, timber harvesting, coal mining, and energy exploration and development [44]. Roads are commonplace and provide human access into grizzly bear habitat, with gravel and secondary roads comprising 96.5% of all roads in potential grizzly bear habitat in Alberta [28]. These roads are also used year-round by the public for a variety of recreational activities.

The Yellowhead region is highly mountainous and land cover, precipitation, and temperature vary from the low elevation valleys to alpine peaks reaching over 3000 m. The natural sub-region classifications for the mountainous areas are alpine and subalpine, and in lower elevations consist of montane, and upper and lower foothills [45]. Coniferous forests dominate, consisting of lodgepole pine (*Pinus contorta*), spruce (*Picea* spp.), and fir (*Abies* spp.). Mixed forests include aspen (*Populus tremuloides*) and balsam poplar (*P*. *balsamifera*, Ladle et. at., 2018). Shrubs, including willow (*Salix* spp.), are scattered throughout the area [25]. Bogs, meadows, and forests regenerating from fire and harvest are also common on the landscape [46]. Due to a history of fire suppression in the foothills, young forests and natural openings are rare [25], whereas large fires have burned through parts of Jasper National Park. The climate is continental, with colder temperatures and higher average annual precipitation in the mountains than in the foothills [47]. Snow cover varies both spatially and temporally due to a mixture of both local and regional climatic conditions [37].

### Grizzly bear data

Global Positioning Systems (GPS) telemetry data from 47 grizzly bears were used from years 2009–2017. Bears were captured during the spring (May-June) using culvert traps and aerial darting from helicopters [48–49]. Followit (Lindesberg, Sweden) GPS radiocollars (Televilt Simplex,and Tellus models) were fitted on captured bears and collected location data at 1 hour intervals. Locations with positional dilution of precision (PDOP) values greater than 10 were removed in order to increase positional accuracy [50]. All grizzly bear captures were authorized under the permitting authority of Alberta Environment and Parks (provincial jurisdiction lands, provincial parks, and protected areas jurisdiction lands), and Parks Canada (federal jurisdiction lands). Research and collection permits were obtained each year from all regulatory agencies. All capture and handling efforts followed guidelines created by the Canadian Council of Animal Care [51] and the American Society of Mammologists [52]. Capture protocols were approved annually by both the University of Saskatchewan’s Committee on Animal Care and Supply and the Alberta Environment and Parks Animal Care Committee.

The period of interest for this research spans from the date of den emergence until May 31^st^ each year, and therefore data used were from the spring following the year of collaring. Data from the 47 bears resulted in 62 bear-years of data, defined as unique years of data from each individual, since some individuals were collared during multiple years. Of the 62 bear-years, 19 were adult (> = 5 years old) females, 6 sub-adult (< 5 years old) females, 3 females with dependants or cubs, 26 adult males, and 8 sub-adult males. Certain individual grizzly bears changed age-reproductive class as their age increased or reproductive status changed.

### Core model covariates

A variety of environmental and landscape covariates were used to characterize grizzly bear habitat selection and use (see Table 1 for a list of all covariates and references to previous work linking variables to grizzly bear selection and use). Data for elevation (ELEV), solar insolation (INSOL), and a topographic wetness index (TWI) were calculated at 30 m spatial resolution from the NASA Shuttle Radar Topography Mission (SRTM) digital elevation model. INSOL represents the amount of primary energy received from the sun, accounting for terrain variation. TWI represents surface water flows and accumulation. These three static variables have been linked to grizzly bear habitat selection [24] and have been used in other step-selection analyses in the study area [32]. An annual landcover classification at 30 m spatial resolution [53] was adapted to represent four distinct classes: forested, forbs, shrubs, and non-vegetated. From the forested class, annual distance to forest edge (dist(FE)) layers were generated, with negative distances representing locations inside of the forest. Road network data was downloaded from the Government of Alberta web portal (https://geodiscover.alberta.ca/) and distance to road (dist(RD)) calculated. All values greater than 1000 m were revalued to 1000 m [54], to account for the diminishing effect of roads at large distances.

The rate of travel of grizzly bears is an important indicator of movement behavior and therefore the natural log of step length (ln(SL)) was calculated from the Euclidean distance between consecutive telemetry locations. Movement of grizzly bears has also been linked to a strong diurnal pattern throughout the day [10,55]. For this reason, time of day (TOD) was calculated at the end of each step to correspond to four periods: dawn (one hour before civil dawn until sunrise), day (sunrise until sunset), dusk (sunset until one hour after dusk), and night (one hour after dusk until one hour before dawn).

### Snow cover variables

**Fig 2:**
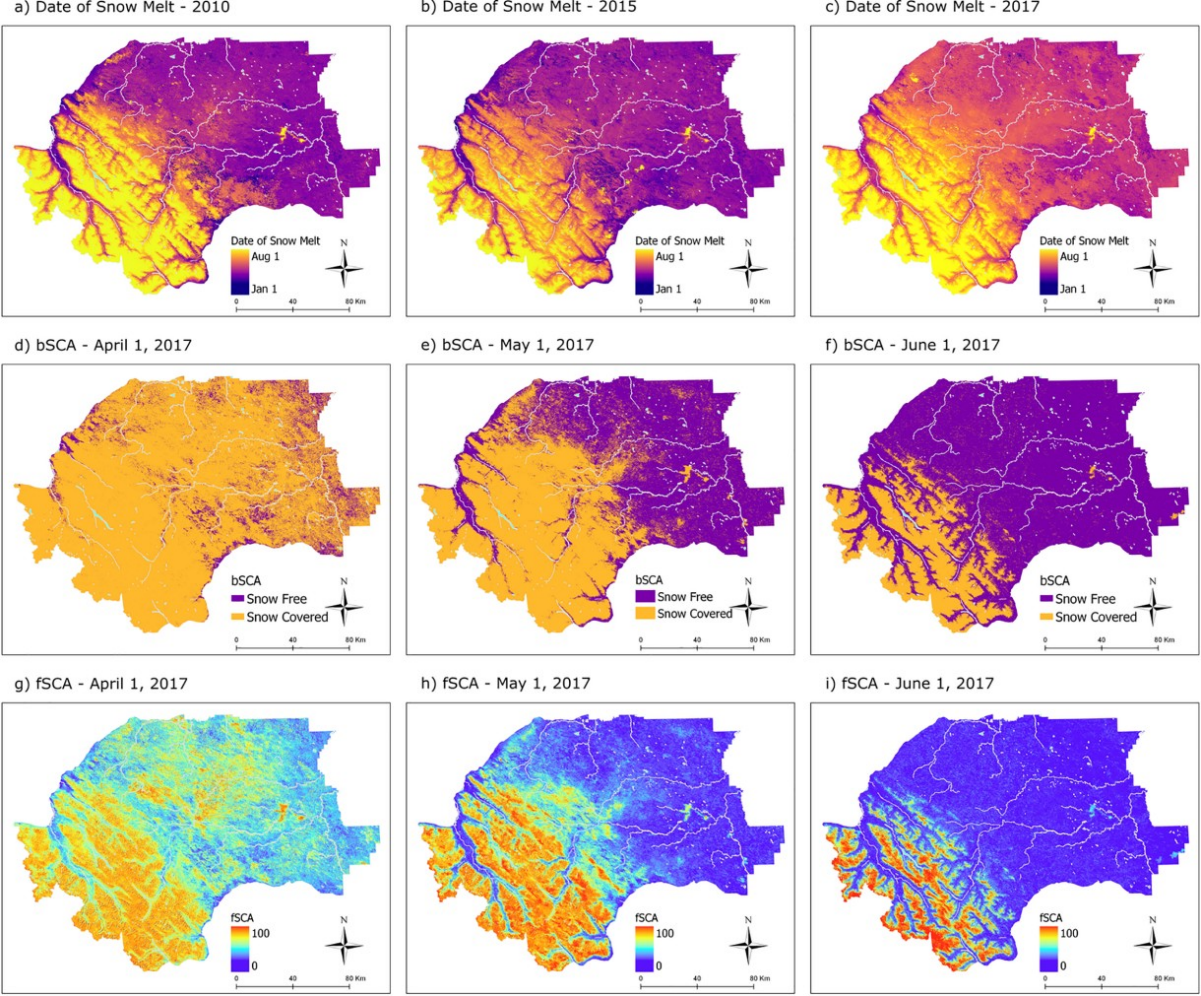
The three snow metrics derived from SNOWARP (Berman et. al., 2018) at 30 m spatial resolution for years 2009–2017. a-c) Annual date of snow melt shown for spring 2010, 2015 and 2017. Days since snow melt (DSM) was produced by subtracting date of snow melt from the date associated with each grizzly bear telemetry location. d-f) daily binary snow covered area (bSCA) shown on April 1, May 1, and June 1, 2017. g-i) daily fractional snow covered area (fSCA) shown on April 1, May 1, and June 1, 2017.

Daily 30–m resolution snow cover data from SNOWARP [37] was used from 2009–2017 (Table 1 and Fig 2). SNOWARP models fractional snow covered area (fSCA), i.e. the percentage between 0–100 of the amount of a pixel that is covered with snow, and is available for a large area of Western Alberta. Using this fSCA dataset, two additional snow variables were derived: binary snow covered area (bSCA) and date of snow melt. bSCA represents snow presence or absence (each 30-m pixel is either “snow covered” or “snow free”), and was calculated using a threshold of 15% on the SNOWARP fSCA product [37]. Date of snow melt is an annual layer, which estimates the date each spring in which a pixel transitions from snow covered to snow free. It was derived from the bSCA dataset by taking the average of a 31-day moving window (15 days before, and 15 days after a given date), and choosing the latest day in the spring (moving backwards from July 31^st^) which had 50% of days “snow covered”, and 50% of days “snow free” within the surrounding window. From the annual date of snow melt layers, daily date since snow melt (DSM) was calculated by subtracting the date of snow melt from the date of each grizzly bear telemetry location (negative values indicating number of days until snow melt, zero indicating the day of snow melt, and positive values indicating the number of days since snow melt).

In addition to using the snow cover variables to model grizzly bear habitat selection and use, the annual date of snow melt layers were used to examine trends in the timing to snow melt. Spatially, the landscape was separated into the 5 natural sub-region classifications (alpine, subalpine, upper foothills, lower foothills, and montane) and for each spring season from 2009–2017 average date of snow melt statistics were extracted by natural sub-region.

### Integrated step selection analysis

Integrated Step Selection Analysis (iSSA) [39–42]is an extension of a large body of work using Resource Selection Functions (RSFs) [25,29,54,56] and Step Selection Functions (SSFs) [31–32,36,57] to model the likelihood that an animal uses an available location, given its resource value quantified through model covariates. SSFs define the domain available to an animal using the empirical distributions of steps taken, where a step is the linear connection of two telemetry locations, defined with the attributes of step length and turning angle [58]. Each step taken is evaluated against a series of randomly generated available steps using conditional logistic regression to determine the likelihood of the animal choosing from available options [59]. The generation of available steps corresponding to a specific time and location allows for the inclusion and evaluation of temporally dynamic variables, such as fractional snow covered area (fSCA) and days since snow melt (DSM). Whereas traditional SSFs exclude movement parameters in the models, iSSA includes either step length or turning angle or a combination of the two, which allows for simultaneously estimating movement and selection parameters [39]. Our implementation of iSSA is described below.

First, grizzly bear location information was transformed into used steps, distinguished by time, location, step length and turning angle. Step length is the Euclidean distance between two consecutive telemetry locations registered at a regular interval [60]. Turning angle is the angular change in direction between steps. Three consecutive telemetry locations are required to calculate turning angle, and therefore steps were only generated for three or more linked locations [58]. Over the 62 bear-years from 2009–2017, 36,645 steps were created to analyze during the period of interest.

Second, five available steps were generated for each used step using a gamma distribution for step length and a von Mises distribution using maximum likelihood for turning angle, both fit from distributions built upon the used steps from each individual bear-year [39]. Once all used and available steps were created, environmental, landscape, and movement variables were extracted (Table 1). Spatial variables were extracted from the end location of each step, as opposed to the start location.

Third, four conditional logistic regression models (Table 2) were fit to the data for each individual bear-year, using the used and available steps generated in step 2. The first model fit was a core model, which included variables that have previously been shown to influence bear movement and selection in the spring. These variables included the log of step length, an interaction between log of step length and time of day, elevation, distance to road, terrain wetness index, distance to forest edge, and solar insolation. Both linear and quadratic terms were included in the model for INSOL, TWI, dist(FE) and dist(RD) to account for non-linear relationships.

**Table 2:** Overview of the four models assessed for each of the 62 bear-years. For covariates with a quadratic term included, the linear term was also included. The AIC Tally is a record of the model that had the lowest Akaike Information Criterion score for each bear-year.

Model	Covariates (quadratic terms all include linear term)	AIC Tally	Average AIC Weight
Core	Ln(SL) + ln(SL):TOD + ELEV + dist(RD)^2^ + TWI^2^ + dist(FE)^2^ + Landcover + INSOL^2^	11	0.1460
Days since snow melt (DSM)	Core + DSM^2^	9	0.1508
Binary snow covered area (bSCA)	Core + bSCA + DSM^2^	5	0.1357
Fractional snow covered area (fSCA)	Core + fSCA^2^ + fSCA:ln(SL) + DSM^2^	37	0.5675

By fitting three additional models built by adding various snow indicators to the core model, it was possible to assess whether snow cover variables improved the model fit, and therefore testing the hypothesis predicting snow as an important factor in characterizing spring season habitat selection and use. The “DSM model” included the core model and days since snow melt (DSM). The “bSCA model” included the core model, binary snow covered area (bSCA), and days since snow melt (DSM). The “fSCA model” included all variables from the core model, fractional snow covered area (fSCA), an interaction between fractional snow covered area and log of step length, as well as days since snow melt (DSM). Both linear and quadratic terms were included for days since snow melt in all three snow models and for fractional snow covered area in the fSCA model.

Next, model fit was evaluated for each model and each individual bear-year to assess the best fitting model and see which of the three snow models, if any, would outperform the core model. To do this, the Akaike Information Criterion (AIC) was calculated for each model run and the one with lowest AIC for each bear-year received a tally, resulting in 62 total tallies. In addition, the average AIC weight [61] was calculated for each model by taking the mean of the AIC weight from each model run. The AIC tally and average AIC weight were used to select the best candidate model, and subsequently that model was used to calculate probability of use.

Probability of use is used to visualize the average effect of covariates, or resource types (such as fSCA) on the probability of space use by grizzly bears [62]. It was generated by computing the predicted probability of selection values of the fitted model outputs from each individual bear-year over all available steps, and smoothing the results using a cubic spline function with 4 knots and 95% confidence intervals. Since the curve was fit over all available points, it represents the average probability of selection conditional on the availability of all resources, and therefore represents the probability of use [62–63]. Probability of use was calculated using the fSCA model in response to both fSCA and DSM. Additionally, data points were stratified by elevation categories (from the ELEV layer) and distance to road in response to DSM. In all analyses DSM was truncated at -50 and 50 to restrict analysis to the period of transition.

Organization of data, model fitting, and analyses were undertaken using the *AMT* (version 0.0.5.0) [64] package in R (version 3.5.1) [65]. ArcGIS Pro (version 2.2.3) [66] was used to pre-process model variables.

## Results

**Fig 3:**
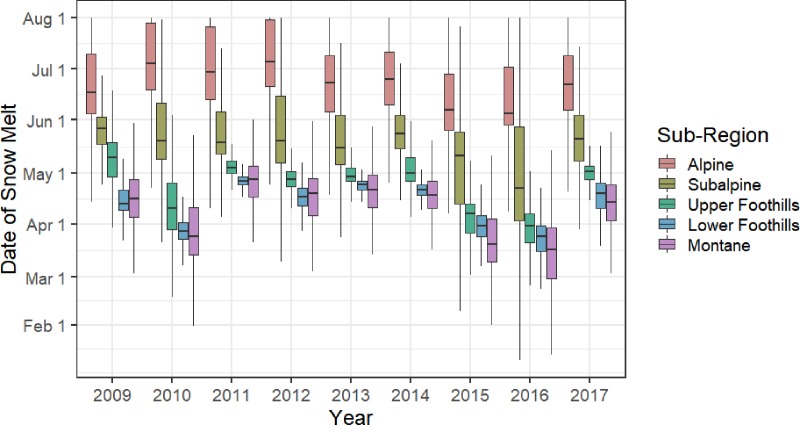
The date of snow melt annually by natural sub-region classification.

The average trends in the date of snow melt on the landscape are shown in Fig 3. Overall, snow in alpine environments melted the latest, whereas snow in montane and lower foothills melted earliest. The timing of snow melt at lower elevations, in upper foothills, lower foothills, and montane environments, fluctuated more year-to-year than at higher elevations in alpine and subalpine regions. Additionally, the years of 2010, 2015, and 2016 can be characterized as years with early snow melt in lower elevation areas.

Through the process of fitting the four candidate models to the individual bear-years, the fSCA model received 37 out of 62 AIC tallies (Table 2), indicating the importance of snow variables in explaining spring movement and selection. The core model received 11 tallies, which is indicative of the variation amongst individual bears and the covariates that influence their selection. The bSCA and DSM models received 5 and 9 tallies respectively, underlying the importance of fine-scale fractional snow mapping (from the fSCA model) when compared to more coarse indicators of snow dynamics. In terms of AIC weight, the fSCA model also significantly outperformed the other models. By dividing the AIC weights, we can determine that the fSCA model is 3.89 times more likely to be the best model than the core model, and 3.76 times more likely to be the best model than the DSM model, the next best performing model that includes snow variables [61].

Comparing the number of AIC tallies received by each model per year (Table 3), a disproportionally high number of tallies were received by the core model for the early snow melt years of 2010, 2015, and 2016. Out of 11 tallies received by the core model, 72.8% were for early snow melt years, whereas the total number of tallies for the early snow melt years account for only 54.8% of the total tallies from 2009–2017.

**Table 3:** A summary of the number of tallies received by each model per year.

Tallies received/year	Core	DSM	bSCA	fSCA
2009	2	0	1	2
2010	1	1	0	2
2011	0	0	0	1
2012	0	0	0	1
2013	0	0	0	3
2014	0	0	0	6
2015	3	2	3	3
2016	4	2	1	11
2017	1	4	0	8

**Fig 4:**
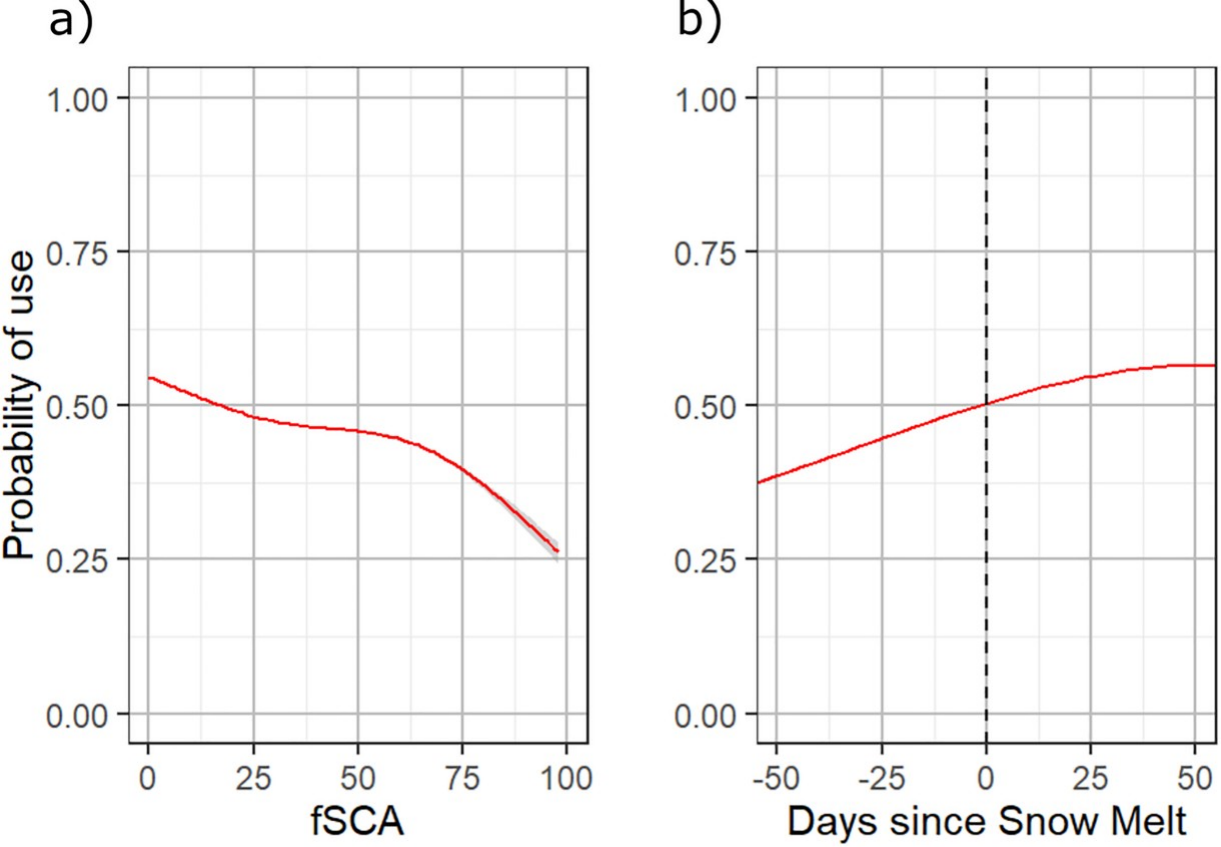
Probability of use was calculated over all available locations with both fSCA and DSM on the x-axis. The overall trends show increased selection for locations with less snow cover than what is available, as well as preference for locations where snow melted sooner in the spring.

**Fig 5:**
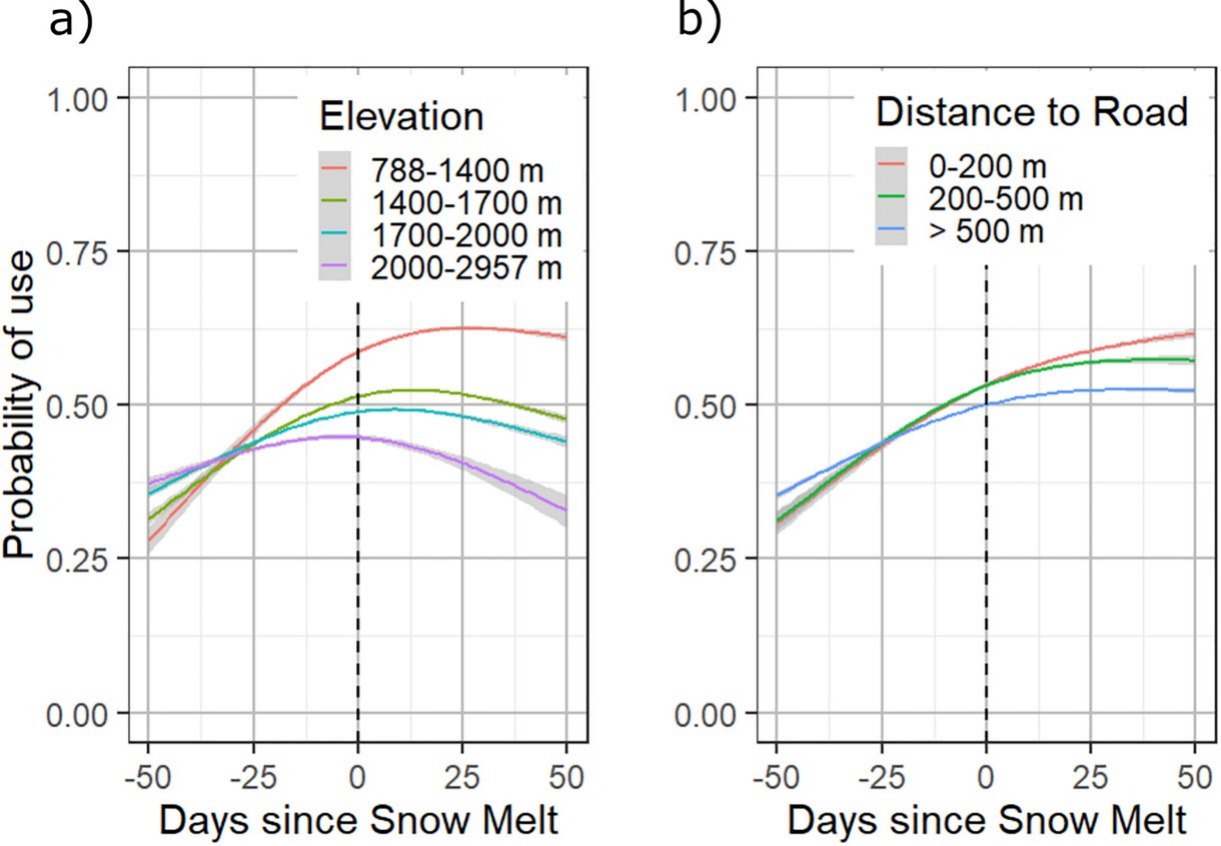
**Probability of use calculated with DSM on the x-axis and (a) categorized by elevation (m) and (b) distance to road (m).** Preference is shown for lower elevation locations, especially once snow has melted. Grizzly bears generally were more likely to select for locations closer to roads once snow has melted.

Probability of use was explored in response to various snow indicators from the top performing model (fSCA model). In response to fSCA (Fig 4A), there was a strong overall correlation between increased probability of use and lower percentages of snow cover. Probability of use was most negatively affected at high percentages of fSCA, between 60–100%. In response to DSM (Fig 4B), probability of use increased as the number of days since snow melt increased. In response to DSM and stratified by elevation (Fig 5A), the effect of DSM was stronger at lower elevations (steeper curve with higher probability) than at higher elevations (flatter curve with lower probability). Use did not vary significantly by elevation prior to snow melt, and reached a maximum effect between 10–25 days after snow melt. When probability of use was tested in response to DSM and stratified by distance to road, a similar trend was present in each distance category, yet locations closer to roads were preferred (Fig 5B).

## Discussion

This work has demonstrated the application of fine scale daily remote sensing data in evaluating a hypothesis relating snow cover variables to spring habitat selection and use of grizzly bears in Western Alberta. Based on the AIC tally and average AIC weights, the inclusion of fSCA improved our predictive model over both a core model and other models which contained more coarse spatial and temporal representations of snow on the landscape. Temporally dynamic covariates such as snow depth have previously been introduced into iSSA [41], however these were shown to demonstrate a weak or variable response, possibly due to low spatial resolution. The advancement of both the resolution and reliability of fine scale remote sensing datasets, such as daily fSCA values at 30 m spatial resolution, hold promise in investigating a range of hypotheses related to wildlife habitat selection, movement, and use. Future applications of this specific dataset include an analysis of grizzly bear denning location and date of den entry and emergence, which has been linked to snow dynamics and food availability [5].

The key findings of this study support the hypothesis that during spring (den emergence to May 31^st^) grizzly bears display preference for using locations with less snow cover (Fig 4A), as well as locations where snow melted sooner (Fig 4B). When bears emerge from denning, spring food resources are often “locked” in the snowpack and are not available until snow melt occurs and the ground thaws [14]. The exception is when bears find or kill ungulates in the spring, which might provide reason to stay in a snow covered location [55]. Our results further validate studies focused on the availability of spring food resources in relation to the probability of bear occurrence [47,67]. Areas where snow melts sooner in the spring provide the first opportunities for feeding, specifically in this study area, where digging for the root *Hedysarum* provides the main staple of the spring diet 55. Earlier snow melt can also result in earlier emergence of other vegetation consumed by bears, and these locations with early food sources are also likely to attract prey species, such as deer and other ungulates.

The results in Table 3 provide insights into why the core model outperformed the snow models in certain instances. The core model received a disproportionately high number of AIC tallies during the years with earliest snow melt (2010, 2015, and 2016). When snow melts earlier on the landscape, it could be a less important factor determining grizzly bear habitat selection during spring, and therefore the variables in the core model would be sufficient for modelling selection. Future analyses may further stratify the landscape based on year-to-year snow melt patterns within individual grizzly bear home ranges, to examine how bears use their home-ranges in different years with regards to snow dynamics.

The results in Fig 5 demonstrate how fine-scale modelling can lead to a better understanding of grizzly bear use of the landscape. When probability of use is stratified by elevation (Fig 5A), grizzly bears show a strong preference for use of lower elevation locations where snow has already melted. Negative human-bear encounters are the most important factor influencing grizzly bear survival in the study region [33,44]. These results link spring snow melt dynamics to the probability of bears using lower elevation locations, which are also the locations that have higher levels of human use. In addition, road networks have strong links to human-caused grizzly bear mortality, especially within 500 m [33,44]. We were interested to know if bears were more likely to use locations closer to roads when snow was present or absent. We found that locations closer to roads (between 0–500 m) were more preferable after snow melted (Fig 5B), possibly due to food availability along road edges, which may melt sooner than other locations. The probability of bears using locations closer to roads is also linked to elevation, as road networks are denser at lower elevations.

One limitation of this study was that grizzly bear telemetry locations were only evaluated at an hourly rate. Effects of snow dynamics on grizzly bear habitat selection probably occur at a variety of temporal scales, including both sub-hourly locations and broader trends in home-range usage inter-annually and throughout different seasons [23]. In addition, data on snow depth was not included, since the spatial resolution of available data was too coarse to match the other fine-scale data of this study. Snow depth can influence activity levels in bears during den entry and emergence periods [11] and in the future could be compared to spring-time resource selection and movement rates. Date of snow melt is potentially a proxy for snow depth, as snow will melt later in locations with greater snow depth, especially if other environmental and terrain factors are accounted for. Finally, we did not compare selection patterns between age-sex-reproductive classes or within individuals collared for multiple years, and it is possible that the magnitude of selection response to snow dynamics my vary depending on the age, sex, and reproductive status of grizzly bears, or be relatively consistent for an individual across different years.

These results have important management implications for bear conservation related to future climatic projections and human-bear encounters during spring in snow free areas. Future climate predictions suggest that winters will have a higher level of uncertainty, with more inter-annual variation in both the timing and extent of winter conditions [18]. In addition, projections show a decline in the number of days with snow cover [20,68–69] and warmer winter and spring temperatures [22]. As winter and snow conditions change, bears will adapt. One implication of changing winter conditions could be a shorter denning season, meaning bears would be present on the landscape for a longer period of time in the spring. This could increase the risk of negative human-bear encounters [5], largely dependent on vegetation dynamics. Worldwide, humans are the main cause of grizzly bear mortality [70]. If lower elevation locations are preferred by bears during this time of year due to snow melt dynamics, earlier snowmelt could also be driving bears into locations with higher risk of human-caused mortality. Future work should apply fine-scale snow maps to develop probability of bear occurrence layers during spring snow melt. These layers could help to identify key locations where snow consistently melts sooner, and these areas could than be designated as target areas for grizzly bear conservation initiatives, such as seasonal access closures to roads and trails.

## Conclusion

Snow conditions are an integral habitat component for a number of species, including grizzly bears, wolverine [71], elk [72], deer [36], and caribou [73]. The inclusion of fine scale snow data has potential to increase our understanding of the interaction between these species and the environment during key times of year. Through the use of iSSA, we have shown daily 30 m fractional snow covered area and annual days since snow melt to improve spring habitat selection models and establish relationships between grizzly bear use of the landscape and snow dynamics. Grizzly bears displayed a strong preference for use of locations with less snow cover in the spring, and areas where snow melted sooner. A better understanding of how bears use the landscape in relation to changing environmental and climatic variables can help resource managers and policy-makers to maintain a sustainable grizzly bear population in both the present as well as the future.
